# Spider Community Variability and Response to Restoration in Arid Grasslands of the Pacific Northwest, USA

**DOI:** 10.3390/insects12030249

**Published:** 2021-03-16

**Authors:** Lauren A. Smith DiCarlo, Sandra J. DeBano

**Affiliations:** 1 Department of Environmental Science, Westfield State University, Westfield, MA 01086, USA; 2 Department of Fisheries and Wildlife, Oregon State University, Hermiston, OR 97838, USA

**Keywords:** Araneae, biodiversity, disturbance, environmental variables, grassland restoration, invasive grasses, litter, old-field restoration, seeding, spider assemblages, vegetative structure

## Abstract

**Simple Summary:**

Spiders contribute greatly to biodiversity and play important roles in terrestrial food webs, yet we know very little about the types of spiders that live in semi-arid grasslands in the Pacific Northwest, USA. As interest in the conservation of biodiversity and restoration of these imperiled ecosystems grows, it is imperative that we not only describe spider communities (i.e., groups of interacting species) in these areas, but also investigate environmental factors that influence them, and how they respond to restoration. This information can help guide land managers as they design restoration and management plans. Our goals were threefold: (1) describe variability in spider communities in three semi-arid grasslands of the Pacific Northwest, (2) identify environmental variables that may influence spider communities, and (3) determine whether spiders or environmental variables differ between actively vs. passively restored sites. We found that spider communities varied greatly among three superficially similar locations and patterns corresponded to differences in elevation and cover of invasive grass, litter, biological soil crust, and flowering plants. We found no differences between spider communities in active and passive restoration treatments, indicating that if the primary goal of restoration is to increase biodiversity, additional factors must also be manipulated.

**Abstract:**

Grassland restoration in North America has intensified but its impact on major invertebrate groups, including spiders, is unclear. We studied three grassland locations in the Pacific Northwest, USA, to (1) describe variability in spider communities, (2) identify environmental variables that may underlie patterns in spider communities, and (3) determine whether spiders and environmental variables differ between actively (removal of disturbances, then plant with natives) vs. passively restored sites (removal of disturbance only). We found spider richness, diversity, and composition differed among the three locations but abundance did not. Sites with more litter and invasive grass cover had more spiders while sites at higher elevation and with more forb and biological soil crust cover had increased spider richness and diversity. Spider community composition was associated with elevation and litter cover. Surprisingly, no spider community or environmental variables differed between actively and passively restored sites, except that litter cover was higher in passively restored sites. This study demonstrates that even in superficially similar locations, invertebrate communities may differ greatly and these differences may prevent consistent responses to active vs. passive restoration. If increasing biodiversity or the abundance of invertebrate prey are goals, then environmental factors influencing spider communities should be taken into account in restoration planning.

## 1. Introduction

Since European settlement, approximately 99% of grasslands in North America have been converted to other land uses, primarily agricultural [1,2]. However, since the late-1800s cropland abandonment has also increased exponentially across North America due to a number of factors including increased urbanization and the import of food from other areas [3]. In response to this trend, there has been a growing movement to restore abandoned fields once used for production to native habitat that can provide essential wildlife habitat and conserve biodiversity [3,4,5,6,7,8]. This effort encompasses arid and semi-arid grasslands of North America with multiple groups, including government agencies and non-profit organizations, at the forefront of restoration. Yet monitoring the effectiveness of restoration efforts in grasslands is limited, often because of lack of funding and data on ecologically significant taxa in many grassland types, including which taxa make up communities and the environmental variables that structure those communities [9,10]. This is particularly true of invertebrates, which make up the majority of grassland biodiversity and provide essential ecosystem services such as pollination, nutrient cycling, food for vertebrates, and pest control [11,12,13,14,15].

One particularly significant group of beneficial invertebrates is spiders, which are the seventh largest taxon in the world with approximately 49,200 known species [16,17]. They not only make major contributions to biodiversity, but also play sizeable roles in food webs as predators and as prey for wildlife [18]. In addition, spiders respond quickly to changes in the surrounding environment and because they are highly mobile and reproduce quickly, they are a useful indicator taxon for restoration monitoring [19,20,21].

Very little is known about grassland spider communities in the US, particularly in the Pacific Northwest. While a few studies have assessed communities in forests e.g., [22,23] and sagebrush steppe e.g., [24,25] only three studies have examined spider communities in grasslands in the region [21,26,27]. Thus, there is a pressing need to describe grassland spider communities and their variability in the Pacific Northwest to help land managers prioritize which grasslands to conserve and restore, and to inform the design of restoration projects.

Studies in other parts of the world that have assessed how grassland restoration affects spiders found mixed results. For example, while multiple studies have found that grassland restoration affects spider abundance, richness, and diversity [21,28,29,30,31], the direction of effects vary, with some studies finding positive effects and others finding negative ones. Other studies have found no effect of restoration on spider abundance, richness, and diversity [28,30,32,33,34]. Furthermore, while many studies have found changes in spider community composition after restoration or grassland succession [21,29,30,32,33,35,36], patterns of response differ from study to study. Several factors may underlie these inconsistent responses, including restoration project parameters (e.g., age, type of plantings), historical factors, and environmental variables known to influence spider abundance, richness, diversity, and community composition (e.g., plant community composition, grass and litter cover) [21,28,29,30,32,37,38,39]. Variability in response demonstrates that the influence of restoration on grassland spider communities may be highly context-dependent [40,41]. The factors contributing to context-dependent responses, and how they vary within a region, may provide insights into spider communities responses to restoration in arid grassland systems. Only one prior study has assessed how Pacific Northwest arid grassland spider communities respond to grassland restoration, at one location [21]. They found that actively restored sites (i.e., removal of disturbance including cultivation and grazing, then seeding with native vegetation) had more spider species than passively restored sites (i.e., removal of disturbance only).

To investigate variability in Pacific Northwest grassland spider communities and responses to restoration, we conducted a study at three separate semi-arid bunchgrass prairies in eastern Oregon that may be expected to show similar responses to old-field restoration. Our objectives were to: (1) describe variability in spider communities at these locations, (2) identify environmental variables associated with patterns in spider communities, and (3) determine whether spider communities or environmental variables consistently differ between actively vs. passively restored sites at the three locations. Given the similarity of the grassland locations, we expected spider communities to respond to restoration in a similar manner and, given previous work in the region [21], that active restoration would result in more species of spiders than passive restoration, and that these changes would be closely associated with differences in litter cover and vegetative structure.

## 2. Materials and Methods

### 2.1. Study Locations

**Figure 1:**
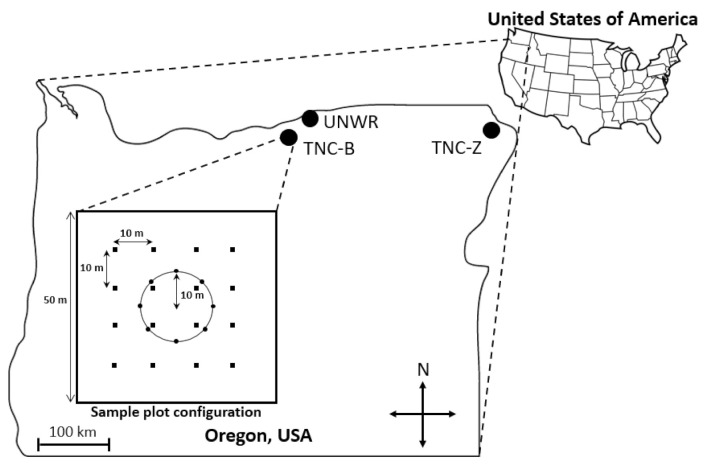
Map of the three study locations (TNC-B, TNZ-Z, and UNWR) in northeastern Oregon, USA with inset showing sample 50 m plot survey configuration. Circles represent pitfall traps; squares represent habitat survey subplots.

The study took place at three grassland locations in Oregon, USA: the Umatilla National Wildlife Refuge (UNWR) and The Nature Conservancy Boardman Grasslands Preserve (TNC-B) in Morrow County, and The Nature Conservancy Zumwalt Prairie (TNC-Z) in Wallowa County (Figure 1). Site characteristics are listed in Table 1. Both TNC-B and TNC-Z contain extensive areas of high-quality grassland (largely intact native grasses and forbs relatively uninvaded with non-native grasses) and all three locations contain passively restored grassland (formerly cultivated with no active effort to restore after disturbance was removed) and actively restored areas (formerly cultivated with herbicide treatment and planting of native vegetation). Cultivation no longer takes place at TNC-B, TNC-Z, and UNWR; however, livestock grazing by cattle (*Bos tauras*) does occur at TNC-Z at low to moderate rates. No grazing occurs at TNC-B or UNWR. In addition, all three locations are exposed to grazing by native ungulates. Mule and white-tailed deer (*Odocoileus hemionus* and *O. virginianus*), elk (*Cervus elaphus*) and pronghorn (*Antilocapra americana*) occur in the region’s grasslands. UNWR and TNC-B have similar invasive and native grass species, while TNC-Z has some similar native species but different invasive grass species (Table 1). The majority of precipitation at UNWR and TNC-B falls from November to February and the majority of precipitation at TNC-Z falls in June [42,43]. Temperatures frequently reach 30 °C in the summer at all locations [43].

### 2.2. Site Selection

Sites were established at each location from three treatments of interest: intact native habitat (control) and passively and actively restored areas. In total, 30 sites were selected, with six sites at UNWR (three actively restored, and three passively restored; no native sites were available); 18 sites at TNC-B (six native, six passively restored and six actively restored), and six sites at TNC-Z (two native, two passively restored and two actively restored). Sites ranged from 129 m to 15,699 m to the nearest site, with an average distance of 639 m between sites. We chose sites by locating habitat that were accessible and that were on relatively flat slopes. Actively restored sites were located in established restoration areas at each location. Actively restored sites had been first treated with herbicides (glyphosate or imazapic) to remove undesirable species, with some receiving a combination of prescribed burning and herbicides (Table 1). Sites were then seeded in the late fall with native bunchgrasses using a range drill. Restoration projects were not irrigated and ranged in size from 4–16 ha at UNWR, 23–42 ha at TNC-B, and 20 ha at TNC-Z.

### 2.3. Spider Sampling

Sampling and surveying followed protocols used in prior work in the area [21]. At each site, eight pitfall traps were placed in a 10 m radius circle (Figure 1). Pitfall traps, 470 mL plastic cups filled with propylene glycol (considered less toxic to non-target wildlife than the preservative ethylene glycol [44]), and placed flush with the soil, are well-suited for collecting ground-active spiders [45]. Spiders were collected at all sites three times in 2015 during three bouts in June–July, July–August, and August–September by opening traps for one week each time period to collect invertebrates. After one week, traps from each site were collected, combined, and transported to the laboratory. Samples were then washed over a 250 μm sieve and spiders sorted from other invertebrates and debris and preserved in 70% ethanol. All juvenile spiders were identified to the family level and all mature spiders were identified to species, if possible, using the keys listed in Table S1.

### 2.4. Habitat Survey

To determine which habitat characteristics were related to spider activity-density (a measure of abundance and spider movement [46], hereafter referred to as abundance), richness, diversity, and species composition, each site was surveyed for environmental variables three times in 2015, coinciding with spider sampling. Variables were estimated to the nearest 5% cover in sixteen 63 × 63 cm subplots located in a 50 × 50 m square around pitfall traps (Figure 1). Variables estimated included the percent cover of invasive grasses, biological soil crusts (algae, cyanobacteria, lichens, microfungi, and mosses found on the soil surface in semi-arid and arid ecosystems), litter, and forbs. We also estimated maximum vegetation height by measuring the tallest stem in each subplot.

### 2.5. Analyses

We calculated spider abundance at each site as the average number of spiders per pitfall because not all pitfalls were present during collection due to weather or animal tampering. Abundance and diversity data were averaged over the three sampling bouts in 2015. All individuals were used to quantify abundance but only mature individuals were used to estimate species richness and diversity (juvenile spiders cannot be positively identified to genus or species without introducing error). We used the Shannon–Weiner index to quantify spider diversity and Chao 1 richness (using EstimateS, Version 9.1.0) to quantify rarified species richness (because samples varied in abundance) [47,48]. Environmental variables for each site were calculated by first averaging subplots for each site and then averaging over the three sampling bouts.

To address our first objective, we used one-way analysis of variance (ANOVA) to determine whether spider abundance, rarefied richness, and Shannon–Weiner diversity differed among locations. All univariate analyses were completed in RStudio 1.0.153 [49]. To compare spider community composition among locations, we used PC-ORD Software version 7.287 for community analyses [50]. Family level abundance was used in all multivariate analyses instead of genus or species abundance to allow for use of juvenile spiders in the analysis. The family dataset contained average spiders per pitfall trap (30 sites × 14 families). The environmental dataset contained environmental variables (30 sites × 6 variables) including: elevation, average maximum vegetation height and average percent cover of invasive grasses, litter, biological soil crusts, and forbs. Data met all statistical assumptions and were not transformed.

Non-metric multidimensional scaling (NMS) with Sorensen distances was used to ordinate sites in the spider family space matrix and the environmental matrix. NMS does not assume linearity between family response and environmental gradients and exposes relationships between the family matrix and the environmental matrix [51]. NMS was performed with 250 random starts and ties were not penalized. A randomization procedure was included to test if solutions were stronger than those obtained by chance, resulting in *p*-values. R^2^ values were calculated to represent the percent variance explained by each axis, and relationships of each axis with spider families and habitat variables were quantified with Pearson correlation coefficients. Multi-response permutation procedures (MRPP) were used with Sorensen distances to test for differences in family composition across locations (UNWR, TNC-B, TNC-Z). Pairwise comparisons resulted in A-statistics, the chance-corrected within-group agreement, and *p*-values.

To address our second objective, we used one-way ANOVAs to identify environmental variables that differed among locations, and we used regression trees and correlations of environmental variables with NMS axes to investigate the relationship of environmental variables with spider variables over all 30 sites. Regression tree analysis is a powerful approach suitable for examining relationships between multiple, potentially interacting, independent variables and a dependent variable [52]. Regression trees repeatedly divide data into two or more groups, each as homogenous as possible, and then divides each of the resulting groups in the same manner, in an iterative process that continues until pre-specified stopping criteria are met. We used a least-squared loss function, which minimizes the sum of the squared deviation. For stopping criteria, we specified a maximum number of splits as 10, a minimum proportion reduction in error (PRE) of 0.05, a minimum split value of 0.05, and a minimum count allowed at any node of 5. We included elevation, average maximum vegetation height, and average percent invasive grass, litter, biological soil crusts, and forb cover as potential predictor variables, and constructed separate trees for spider abundance, rarefied richness, and diversity. Regression tree models were conducted using SYSTAT v. 13 [53].

To address our third objective, we used a subset of 22 sites at the three locations that were either actively or passively restored to determine if spider communities at actively restored sites consistently differed from those at passively restored sites, regardless of location. To compare average spider abundance, richness, rarefied richness, diversity, and environmental variables in passively and actively restored sites, we used one-way ANOVAs, blocked by location. We used MRPP to compare spider composition between actively and passively restored sites.

## 3. Results

A total of 1595 spiders were collected from the 30 sites at the three locations. Spiders belonged to 14 families and 35 identified species (Table S1). Five spider families contributed 95.7% of all spiders collected (Theridiidae: 38.6%; Lycosidae: 22.9%; Gnaphosidae: 17.2%; Thomisidae: 11.8%; Salticidae: 5.2%). Of individuals collected, 21% were mature, 69% were immature, and the remaining individuals’ maturity was unknown due to bodily damage. We collected 299 individuals at UNWR, 1046 at TNC-B, and 250 at TNC-Z.

### 3.1. Objective 1

Spider rarefied richness and diversity significantly differed among the three locations; however, spider abundance did not (Table 2). Spider rarefied richness was highest at TNC-Z but did not differ between TNC-B and UNWR (Table 2). Spider diversity was highest at UNWR and TNC-Z, which did not significantly differ from each other, but did differ from TNC-B (Table 2).

**Figure 2:**
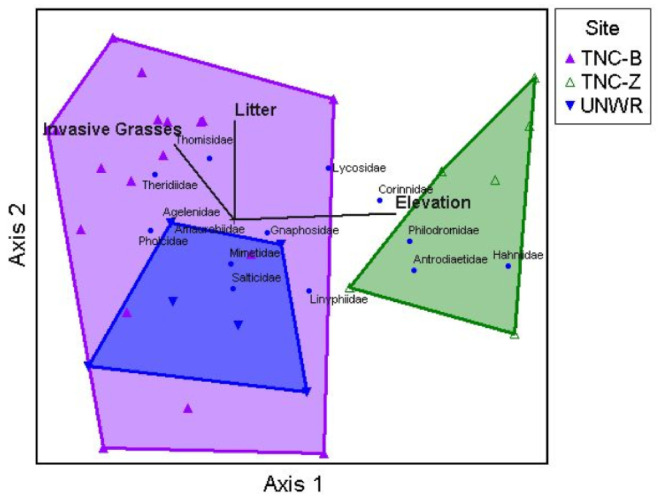
Non-metric multidimensional scaling ordination of sites in spider family space along with weighted average positions for spider families for axes 1 vs. 2. Each triangle represents a site and each blue circle point represents a spider family. A joint-plot from the environmental matrix is overlaid with variables of r^2^ > 0.30 being displayed with vector lengths corresponding to the correlation strength along the axes (shown in thick black lines). Sites that are closer together are more similar than sites farther away from each other. Convex hulls connect each group of sites relative to location.

The NMS randomization procedure revealed that spider community composition varied among locations, resulting in a stable three-dimensional solution (final stress = 7.85, final instability = 0, *p* = 0.01) with a cumulative R^2^ of 0.95. Axis 1 accounted for 56.6% of the variation in spider family space, axis 2 accounted for 28.7%, and axis 3 accounted for 10.1%. Spider community composition did show patterns relative to location (Figure 2) and MRPP analysis showed community composition differed significantly among locations (A = 0.20, *p* < 0.00001). Pairwise comparisons suggested that communities at TNC-B and UNWR differed from TNC-Z (A = 0.21, *p* = < 0.00001 and A = 0.22, *p* = 0.0009, respectively) and also from each other (A = 0.06, *p* = 0.01). Separation among locations occurred on both axes. Pearson correlations between the three axes and spider taxa and environmental variables are listed in Table 3. TNC-B and UNWR sites differed from TNC-Z along axis 1, with TNC-B and UNWR completely overlapping on the left side of the ordination (Figure 2) while TNC-Z (located on the right side of the ordination) did not overlap with either site. Lycosidae (wolf spiders) and Philodromidae (philodromid crab spiders) were all positively correlated with axis 1 and associated more with TNC-Z sites. Theridiidae (cobweb spiders) was negatively correlated with axis 1 and was more common in TNC-B and UNWR. Only Thomisidae (crab spiders) was positively correlated with axis 2 and thus TNC-B and TNC-Z. Antrodiaetidae (folding trapdoor spiders) was positively correlated with axis 3 and TNC-Z, while Gnaphosidae (ground spiders) and Salticidae (jumping spiders) were negatively correlated with axis 3 and more common in TNC-B and UNWR. As axis 3 only explains approximately 10% of the variation, it will not be discussed further.

### 3.2. Objective 2

Several environmental variables differed significantly among the three locations. Invasive grass cover was significantly higher at TNC-B compared to TNC-Z, with levels of cover at UNWR intermediate between the two (Table 2). Maximum vegetation height was taller at TNC-Z than TNC-B, with vegetation height at UNWR intermediate between the two, and forb cover was higher at TNC-Z and UNWR than TNC-B (Table 2). Litter and biological soil crusts did not differ significantly among locations (Table 2).

**Figure 3:**
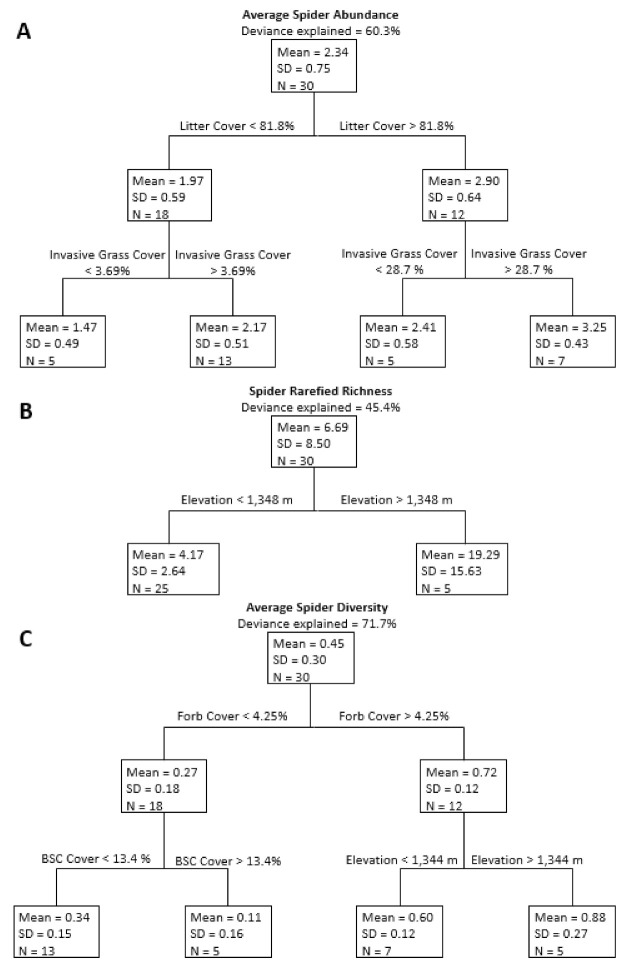
Regression trees for average spider abundance (**A**), rarefied richness (**B**), and diversity (**C**) using each study site. Deviance explained is the amount of variability explained by the full regression tree. Variables associated with each branch are above the horizontal lines above the nodes. Each node (box) of the trees includes mean response variables (spider abundance, rarefied richness, or spider diversity); standard deviation (SD); and sample size (N).

When investigating the relationship between environmental variables and spider response variables over all 30 sites, regression tree analysis for spider abundance produced a tree with four terminal nodes that explained approximately 60.3% of the variation in abundance (Figure 3A). The first two branches formed relative to litter cover, with sites with high litter cover having more spiders than sites with lower litter cover. Sites with high and low levels of litter both branched into two terminal nodes based on invasive grass cover, with sites with less invasive grass cover having fewer spiders than sites with more invasive cover. Regression tree analysis of spider rarefied richness produced a tree with two terminal nodes that separated relative to elevation and explained 45.4% of the variation (Figure 3B). Lower elevation sites had lower species richness than higher elevation sites. Regression tree analysis of spider diversity produced a tree with four terminal nodes that explained 71.7% of the variation in diversity (Figure 3C). The first two branches formed relative to percent forb cover, with sites with more forb cover having higher spider diversity. Sites with higher forb cover split into two terminal nodes relative to elevation; sites at higher elevations had higher spider diversity. Sites with less forb cover branched relative to biological soil crust cover, with sites with more biological soil crust cover having lower diversity than sites with less biological soil crust cover.

When investigating the relationship of environmental variables with spider community composition over all 30 sites, only two variables strongly correlated with axes 1 and 2 (Figure 2; Table 3). Elevation was positively correlated with axis 1 and, therefore, was positively related to the abundance of Lycosidae and Philodromidae, and negatively related to the abundance of Theridiidae. Litter cover was positively correlated with axis 2, which was positively related to the abundance of Thomisidae.

### 3.3. Objective 3

When comparing actively and passively restored sites, there were no significant differences in spider abundance, rarefied richness, or diversity (Table 4). Likewise, MRPP analysis found no difference between the spider communities within the actively or passively restored sites (A = −0.01, *p* = 0.65). Of environmental variables, only litter cover differed significantly, with higher amounts of litter within passively restored sites compared to actively restored sites (Table 4).

## 4. Discussion

Spiders are vital to biodiversity and terrestrial food webs, yet we know little of spider communities within semiarid grasslands in the Pacific Northwest, USA. This information has become essential as different groups and organizations push for conservation of biodiversity and restoration within these grasslands. We sought insight into the composition of these spider communities, which factors underlie community patterns, and how these communities respond to different grassland restoration practices.

We found that even in superficially comparable locations, most characteristics of the spider communities differed markedly. Although spider abundance was similar among locations, richness, diversity, and composition differed significantly. We also identified differences in environmental variables among locations, with one grassland location (TNC-Z) having relatively low invasive grass cover, high forb cover, and tall vegetation; one grassland location (TNC-B) having high invasive grass cover, low forb cover, and relatively short vegetation; and the third location (UNWR) having intermediate levels of invasive grass cover and vegetation height, and relatively high forb cover.

Our analyses of environmental and spider response variables at the 30 sites allowed us to identify which variables may be driving patterns in spider abundance, richness, diversity and structure. The site-level analyses showed that litter was strongly related to spider abundance, which corresponds well to the larger pattern at the location-scale, in which the litter cover and spider abundance did not differ among locations. Litter can benefit certain groups of common ground-active spiders (crab and wolf spiders) that rely on litter for hunting [21,26,40] and we did find that litter was associated with composition changes, including a positive correlation with crab spider (Thomisidae) abundance. Crab and wolf spiders collected do not build webs, and these ground-dwelling spiders have been found to prefer higher litter cover while foraging [40,54,55]. Invasive grass cover was also associated with spider abundance, a relationship that may be modulated through litter inputs because invasive grasses can contribute large amounts of ground litter in these systems [26,56].

Site-level analyses identified only one variable associated with observed patterns in spider richness: elevation. This also corresponds with the patterns observed at the location-level; as the magnitude of difference in spider richness between TNC-Z and the other two locations corresponds well to the magnitude of difference in elevation among locations. Other studies have also found that spider species richness is positively correlated with elevation because higher elevation sites tend to have lower summer temperatures and higher plant richness [57,58]. These factors most likely result in an increase of moisture that may benefit many spider families, especially wolf spiders, which prefer areas with higher moisture [59,60]. We found higher elevation sites had more folding trapdoor spiders, corrinid sac spiders, ground spiders, dwarf sheet weavers, wolf spiders, and philodromid crab spiders. Members of the dwarf sheet weaver family are known to build webs near water to condense moisture and remain active during dry times of the day [60,61,62]; however, little is known about the particular folding trapdoor spider genus (*Androdiaetus*) found in our study, as it has only been collected at a handful of locations in the Pacific Northwest including the Blue Mountains of eastern Oregon and species are still being described [63]. Cobweb spiders, the majority of which were immature western black widows (Theridiidae: *Latrodectus hesperus*), were only collected at the lower elevation sites. Cobweb spiders are common generalists associated with arid grasslands and may benefit from the warmer temperatures and drier climate; however, these spiders have been known to occupy multiple types of habitat [21,64].

Site-level analyses identified forb cover as the primary variable associated with patterns in regional spider diversity. Forbs can contribute to spider diversity by increasing habitat complexity through added litter accumulation and increased vegetative structure. This complexity can provide additional habitat for various groups, including web-spinners and floral hunters [18,30,36,65].

Previous work has found that spider communities respond to restoration with changes in abundance, richness, and/or diversity and almost all detect differences in community composition between treatments e.g., [21,28,29,36]. We found no evidence that spider, abundance, richness, diversity, or composition differed between passively and actively restored sites consistently across all locations. Likewise, all environmental variables, except for litter, showed no difference between the two types of restoration treatment. Litter was the only variable that showed a consistent response to active vs. passive restoration at all three locations, with lower levels of litter associated with actively restored sites compared to passively restored sites. A change in litter between passive and actively restored sites is not surprising as passively restored sites discontinued farming and/or grazing, leaving all vegetation intact, including invasive grass species that can create large amounts of litter. Actively restored sites, in addition to discontinuing sources of disturbance, chemically removed the majority of vegetation before seeding with bunchgrasses which create far less litter than the invasive annual grass species. Depending on the restoration goals, intentional activities that impact environmental variables may need to be used to alter spider communities.

Our inability to detect consistent differences between actively and passively restored sites across locations contrasts with earlier work that showed an increase in species richness in actively restored sites compared to passively restored ones [21]. That study was conducted at one of the locations examined in this study during a three-year time period. Patterns of response to active vs. passive restoration for that one location were consistent between studies, suggesting that variation in responses to restoration is more strongly associated with location and less influenced by inter-annual differences in climate. The lack of a consistent effect across locations suggests that location-scale differences may influence the relative advantages of using passive vs. active restoration methods in grasslands. Active methods are more time-consuming and expensive, so future research should focus on identifying the particular characteristics of grasslands that lead to differential responses to passive vs. active restoration.

## 5. Conclusions

As conservation and restoration of biodiversity increases in these rare grasslands, predicting responses to particular restoration efforts, such as passive and active restoration, will depend largely on the knowledge of the spider community and the factors that influence them. We found that even in superficially similar locations, spider communities can differ greatly. Thus, updated local knowledge of the spider communities are key. Combining that information with knowledge of site-specific conditions, such as invasive grass and litter cover, and restoration practices may help maximize the effectiveness of grassland restoration. We encourage further study of the spider communities within arid and semiarid grasslands, given their ecological importance and utility as an indicator taxon.

## Figures and Tables

**Table 1:** Information on restoration sites from the Umatilla National Wildlife Refuge (UNWR) and The Nature Conservancy Boardman Grasslands Preserve (TNC-B) in Morrow County and The Nature Conservancy Zumwalt Prairie (TNC-Z) in Wallowa County, Oregon, including coordinates, size (ha), total number of sites, average annual precipitation (cm) and average annual temperatures (°C) (30-year averages, US Climate Data, 2017), common native grasses, common invasive grasses, elevation (m), year seeded, and type of restoration. Site refers to an active restoration project at each location.

Location	Coordinates	Size (ha)	Total Number of Sites (Passively Restored, Actively Restored, Native)	Average Annual Precipitation (cm)	Average Annual Temperature (low–high °C)	Common Native Grasses *	Common Invasive Grasses *	Actively Restored Sites	Elevation (m)	Year Seeded	Type of Restoration
UNWR	45.905256° N, −119.584475° W	3604	6 (3, 3, 0)	22	5–18	PSSPS; POSE; ELELE; HECOC8	BRTE; TACA8	1	86	2007	Herbicide + Burn
								2	83	2013	Herbicide
								3	84	2015	Herbicide + Burn
TNC-B	45.636738° N, −119.860457° W	9163	18 (6, 6, 6)	19	5–18	PSSPS; POSE; ELELE; HECOC8	BRTE; TACA8	1	267	2006	Herbicide
								2	272	2008	Herbicide
								3	274	2009	Herbicide
								4	281	2010	Herbicide
								5	245	2011	Herbicide
								6	256	2012	Herbicide
TNC-Z	45.555802° N, −116.958538° W	13,354	6 (2, 2, 2)	48	−1–16	PSSPS; POSE; FEID; KOMA	VEDU; THIN6; AGCR	1	1352	2010	Herbicide + Burn
								2	1379	2010	Herbicide + Burn

* AGCR: crested wheatgrass (*Agropyron cristatum*); BRTE: cheatgrass (*Bromus tectorum*); ELELE: bottlebrush squirreltail (*Elymus elymoides*); FEID: Idaho fescue (*Festuca idahoensis*); HECOC8: needle and thread grass (*Heterostipa comate*); KOMA: prairie Junegrass (*Koeleria macrantha*); POSE: Sandberg bluegrass (*Poa secunda*); PSSPS: bluebunch wheatgrass (*Pseudoroegneria spicata*); TACA8: medusahead (*Taeniatherum caput-medusae*); THIN6: intermediate wheatgrass (*Thinopyrum intermedium*); VEDU: ventenata (*Ventenata dubia).*

**Table 2:** Mean (± standard error (SE)) spider abundance, rarefied richness, and Shannon diversity and environmental variables of three grasslands in the Pacific Northwest, and location effects using one-way analyses of variance (ANOVAs). Tukey’s honestly significant difference (HSD) test was used for mean comparisons and superscripts represent significantly different means for the post-hoc tests. Location effects in bold are statistically significant at the *p* < 0.05 level.

Dependent Variable	TNC-B	UNWR	TNC-Z	Location Effect
Spider Abundance	2.47 ± 0.20	2.08 ± 0.21	2.21 ± 0.28	F(_2_) = 0.70, *p* = 0.51
Spider Richness	3.37 ± 0.58 ^a^	6.38 ± 0.86 ^a^	16.9 ± 6.17 ^b^	**F(_2_) = 8.83, *p* < 0.01**
Spider Diversity	0.28 ± 0.05 ^a^	0.60 ± 0.06 ^b^	0.80 ± 0.13 ^b^	**F(_2_) = 14.9, *p* < 0.01**
Invasive Grasses	25.9 ± 4.68 ^a^	21.8 ± 6.33 ^a,b^	3.65 ± 1.64 ^b^	**F(_2_) = 3.78, *p* = 0.04**
Litter	69.9 ± 6.29	60.7 ± 3.33	75.3 ± 5.34	F(_2_) = 0.68, *p* = 0.52
Biological Soil Crust	6.69 ± 2.62	1.66 ± 1.01	1.10 ± 0.80	F(_2_) = 1.28, *p* = 0.29
Forbs	1.31 ± 0.34 ^a^	6.45 ± 0.61 ^b^	5.84 ± 0.73 ^b^	F(_2_) = 36.1, *p* < 0.01
Max Veg Height	29.4 ± 3.52 ^a^	34.7 ± 1.22 ^a,b^	48.6 ± 3.88 ^b^	**F(_2_) = 5.23, *p* = 0.01**
Elevation	252.5 ± 3.52 ^a^	86.7 ± 2.12 ^b^	1362.8 ± 6.73 ^c^	**F(_2_) = 16,551.7, *p* < 0.01**

* All spider variables are average per pitfall per site. All environmental variables are percent cover, except for vegetation height, which is in centimeters, and elevation, which is in meters.

**Table 3:** Pearson correlation coefficients between environmental variables and spider families and each axis of the 3-dimensional non-metric multidimensional scaling (NMS) ordination. Cells with significant correlation coefficients (r^2^ > 0.35) are in bold.

		Axis 1		Axis 2		Axis 3	
		R	R^2^	R	R^2^	R	R^2^
Environmental Variables	Elevation	**0.82**	**0.66**	0.17	0.03	0.24	0.06
	Invasive Grass Cover	−0.50	0.25	0.55	0.31	0.03	0.00
	Litter Cover	0.08	0.01	**0.64**	**0.40**	−0.19	0.04
	BSC Cover	−0.23	0.06	−0.47	0.22	0.07	0.01
	Max Veg Height	0.30	0.09	0.46	0.21	0.05	0.00
	Forb Cover	0.36	0.13	−0.13	0.02	0.02	0.00
Spider Families	Agelenidae	−0.08	0.01	−0.05	0.00	0.10	0.01
	Amaurobiidae	−0.08	0.01	−0.01	0.00	0.10	0.01
	Antrodiaetidae	0.39	0.15	−0.13	0.02	**0.61**	**0.37**
	Corinnidae	0.38	0.15	−0.07	0.00	−0.10	0.01
	Gnaphosidae	0.51	0.26	−0.27	0.06	**−0.59**	**0.35**
	Hahniidae	0.52	0.27	−0.11	0.01	−0.03	0.00
	Linyphiidae	0.23	0.05	−0.27	0.07	−0.21	0.04
	Lycosidae	**0.75**	**0.56**	0.52	0.27	−0.09	0.01
	Mimetidae	−0.01	0.00	−0.13	0.02	0.01	0.00
	Philodromidae	**0.66**	**0.43**	−0.10	0.01	−0.45	0.20
	Pholcidae	−0.19	0.04	−0.03	0.00	0.06	0.00
	Salticidae	−0.01	0.00	−0.43	0.18	**−0.62**	**0.38**
	Theridiidae	**−0.76**	**0.58**	0.53	0.28	−0.09	0.01
	Thomisidae	−0.23	0.05	**0.71**	**0.51**	−0.03	0.00

**Table 4:** Mean (± SE) spider abundance, rarefied richness, and Shannon diversity and environmental variables in passively and actively restored sites located in three grasslands in the Pacific Northwest, and the restoration effect using ANOVA blocked by location. Restoration effects in bold are statistically significant at the *p* < 0.05 level.

Dependent Variable	Passively Restored	Actively Restored	Restoration Effect
Spider Abundance	2.53 ± 0.26	2.59 ± 0.17	F(_1_) = 0.02, *p* = 0.90
Spider Richness	6.44 ± 2.32	5.37 ± 0.63	F(_1_) = 0.23, *p* = 0.63
Spider Diversity	0.47 ± 0.09	0.49 ± 0.05	F(_1_) = 0.10, *p* = 0.76
Invasive Grasses	26.1 ± 4.89	27.19 ± 6.17	F(_1_) = 0.03, *p* = 0.86
Litter	82.9 ± 4.84	69.39 ± 4.95	**F(_1_) = 6.60, *p* = 0.02**
Biological Soil Crust	0.91 ± 0.57	0.36 ± 0.16	F(_1_) = 1.04, *p* = 0.32
Max Veg Height	33.15 ± 3.78	40.54 ± 2.49	F(_1_) = 3.51, *p* = 0.08
Forbs	3.45 ± 0.99	3.57 ± 0.71	F(_1_) = 0.03, *p* = 0.87

* All spider variables are average per pitfall per site. All environmental variables are percent cover, except for vegetation height, which is in centimeters, and elevation, which is in meters.

## Data Availability

The data presented in this study are available in Table S1, other data are available on request from the corresponding author, as they are not published at this time.

## Supplementary Materials

The following are available online at https://www.mdpi.com/2075-4450/12/3/249/s1, Table S1, List of identified spider species including total count and collection locations.
