# Supplementary material for: Spider Community Variability and Response to Restoration in Arid Grasslands of the Pacific Northwest, USA
Source: Insects. 2021 Mar 16;12(3):249. doi: 10.3390/insects12030249 (PMC7998894; doi:10.3390/insects12030249)
Supplement: Supplementary file 1 [file insects-12-00249-s001.pdf]

Table S1. List of identified spider species including total count and collection locations. Bold cells include counts of mature identified species. Unknown species linked to families or genera were largely immature individuals but may include several individuals with some bodily damage that prevented accurate identification. Individuals with unknown family had bodily damage that prevented identification.

<table>
<tr><th>Family</th><th>Genus</th><th>Species</th><th>Authority</th><th>Total Count</th><th>Collection Locations</th></tr>
<tr><td>Agelenidae</td><td>Unknown</td><td>spp.</td><td></td><td>1</td><td>UNWR</td></tr>
<tr><td>Amaurobiidae</td><td>Cybaeopsis</td><td>sp.</td><td></td><td>1</td><td>UNWR</td></tr>
<tr><td>Antrodiaetidae</td><td>Antrodiaetus</td><td>pugnax</td><td>Chamberlin, 1917</td><td>4</td><td>TNC-Z</td></tr>
<tr><td rowspan="2">Corinnidae</td><td rowspan="2">Castianeira</td><td>longipalpa</td><td>Hentz, 1847</td><td>1</td><td>UNWR</td></tr>
<tr><td>spp.</td><td></td><td>3</td><td>TNC-Z; UNWR</td></tr>
<tr><td rowspan="20">Gnaphosidae</td><td rowspan="3">Drassodes</td><td>neglectus</td><td>Keyserling, 1887</td><td>1</td><td>TNC-Z</td></tr>
<tr><td>saccatus</td><td>Emerton, 1890</td><td>1</td><td>TNC-Z</td></tr>
<tr><td>spp.</td><td></td><td>1</td><td>TNC-Z</td></tr>
<tr><td rowspan="2">Drassyllus</td><td>dromeus</td><td>Chamberlin, 1922</td><td>3</td><td>TNC-B; TNC-Z</td></tr>
<tr><td>lamprus</td><td>Chamberlin, 1920</td><td>3</td><td>TNC-B; TNC-Z; UNWR</td></tr>
<tr><td rowspan="3">Gnaphosa</td><td>californica</td><td>Banks, 1904</td><td>17</td><td>TNC-B; UNWR</td></tr>
<tr><td>muscorum</td><td>L. Koch, 1866</td><td>1</td><td>TNC-Z</td></tr>
<tr><td>sericata</td><td>L. Koch, 1866</td><td>2</td><td>TNC-B</td></tr>
<tr><td>Haplodrassus</td><td>hiemalis</td><td>Emerton, 1909</td><td>1</td><td>TNC-B</td></tr>
<tr><td>Nodocion</td><td>voluntaries</td><td>Chamberlin, 1919</td><td>1</td><td>TNC-Z</td></tr>
<tr><td>Urozelotes</td><td>rusticus</td><td>L. Koch, 1872</td><td>1</td><td>TNC-B</td></tr>
<tr><td rowspan="7">Zelotes</td><td>duplex</td><td>Chamberlin, 1922</td><td>2</td><td>TNC-Z</td></tr>
<tr><td>exiguioides</td><td>Platnick & Shadab, 1983</td><td>1</td><td>TNC-Z</td></tr>
<tr><td>fratis</td><td>Chamberlin, 1920</td><td>2</td><td>TNC-Z</td></tr>
<tr><td>josephine</td><td>Platnick & Shadab, 1983</td><td>3</td><td>TNC-Z</td></tr>
<tr><td>puritanus</td><td>Chamberlin, 1922</td><td>44</td><td>TNC-B; TNC-Z; UNWR</td></tr>
<tr><td>sula</td><td>Lowrie & Gertsch, 1955</td><td>1</td><td>TNC-Z</td></tr>
<tr><td>tubuous</td><td>Chamberlin, 1919</td><td>6</td><td>TNC-Z; UNWR</td></tr>
<tr><td>Unknown</td><td>spp.</td><td></td><td>184</td><td>TNC-B; TNC-Z; UNWR</td></tr>
<tr><td>Hahniidae</td><td>Neoantistea</td><td>magna</td><td>Keyserling, 1887</td><td>8</td><td>TNC-Z</td></tr>
<tr><td>Linyphiidae</td><td>Erigone</td><td>dentosa</td><td>O. Pickard-Cambridge, 1894</td><td>1</td><td>TNC-B</td></tr>
</table>

		spp.		1	TNC-Z
	Unknown	spp.		13	TNC-Z; UNWR
**Lycosidae**	***Schizocosa***	***mccooki***	**Montgomery, 1904**	**95**	**TNC-B; TNC-Z; UNWR**
		minnesotensis	**Gertsch, 1934**	**25**	**TNC-B**
		spp.		144	TNC-B
	Unknown	spp.		101	TNC-Z; UNWR
**Mimetidae**	***Mimetus***	***hesperus***	**Chamberlin, 1923**	**2**	**UNWR**
	Unknown	spp.		1	UNWR
**Philodromidae**	***Ebo***	**spp.**		**2**	**TNC-Z**
	Uba	**spp.**		**1**	**TNC-Z**
	Unknown	spp.		6	TNC-B; TNC-Z; UNWR
**Pholcidae**	***Psilochorus***	***hesperus***	**Gertsch & Ivie, 1936**	**1**	**UNWR**
	Unknown	spp.		7	TNC-B; UNWR
**Salticidae**	***Habronattus***	**spp.**		**19**	**TNC-Z; UNWR**
	Pellenes	**spp.**		**4**	**TNC-B**
	Phidippus	***johnsoni***	**Peckham & Peckham, 1883**	**3**	**TNC-B; TNC-Z; UNWR**
	Unknown	spp.		57	TNC-B; TNC-Z; UNWR
**Theridiidae**	***Latrodectus***	***hesperus***	**Chamberlin & Ivie, 1935**	**1**	**UNWR**
		spp.		511	TNC-B
	Asagena	***fulva***	**Keyserling, 1884**	**19**	**TNC-B; UNWR**
		spp.		1	TNC-B
	Unknown	spp.		84	TNC-B; TNC-Z
**Thomisidae**	***Xysticus***	***cunctator***	**Thorell, 1877**	**21**	**TNC-B; TNC-Z; UNWR**
		gulosus	**Keyserling, 1880**	**7**	**TNC-Z**
		spp.		118	TNC-Z; TNC-B
	Unknown	spp.		42	TNC-B; TNC-Z; UNWR
Unknown	Unknown	Unknown		15	TNC-B; TNC-Z; UNWR

*Keys used for identification:

1. Bug Guide. Available online: https://bugguide.net/node/view/15740 (accessed on 8 March 2021).
2. Coyle FA. Systematics and Natural History of the Mygalomorph Spider genus *Antrodiaetus* and Related Genera (Araneae: Antrodiaetidiae). *Bull Mus Comp Zool* **1971**, 141, 269-402.

3. Dondale CD, Redner, JH. *The insects and Arachnids of Canada Part 5: The crab spiders of Canada and Alaska, Araneae: Philodromidae and Thomisidae*. Agriculture, Ontario, Canada, 1978; pp. 1-255.
4. Dondale CD, Redner, JH. *The insects and Arachnids of Canada Part 17: The wolf spiders, nurseryweb spiders, and lynx spiders of Canada and Alaska, Araneae: Lycosidae, Pisauridae, and Oxyopidae*. Agriculture, Ontario, Canada, 1990; pp. 1-383.
5. Edwards GB. *Revision of the jumping spiders of the genus Phiddippus (Araneae: Salticidae).* Occasional Papers of the Florida State Collection of Arthropods, Vol. 11, Gainseville, Florida, USA, 2004; pp. 1-158.
6. Huber BA. New World pholcid Spiders (Araneae: Pholcidae): A revision at generic level. *Bull Am Mus Nat Hist* **2000**, 254, 1-348.
7. Levi HW, Levi LR. The genera of the spider family Theridiidae. *Bull Mus Comp Zool* **1962**, 127, 1-72.
8. Platnick NI, Dondale CD. *The insects and Arachnids of Canada Part 19: The ground spiders of Canada and Alaska, Araneae: Gnaphosidae*. Agriculture, Ontario, Canada, 1992; pp. 1-297.
9. Reiskind J. The spider subfamily Castianeirinae of North and Central America (Araneae, Clubionidae). *Bull Mus Comp Zool* **1969**, 138, 163-326.
10. Ubick D, Paquin P, Cushing PE, Roth V (eds). *Spiders of North America: an identification manual*. American Arachnological Society, Keene, New Hampshire, USA, 2004; pp. 1-377.
